# Langmuir–Blodgett Transfer of Nanocrystal Monolayers: Layer Compaction, Layer Compression, and Lattice Stretching of the Transferred Layer

**DOI:** 10.3390/nano14141192

**Published:** 2024-07-12

**Authors:** Reken N. Patel, Brian Goodfellow, Andrew T. Heitsch, Detlef-M. Smilgies, Brian A. Korgel

**Affiliations:** 1 Department of Chemical Engineering, Center for Nano- and Molecular Science and Technology, Texas Materials Institute, The University of Texas at Austin, Austin, TX 78712, USA; 2 Cornell High Energy Synchrotron Source (CHESS), Cornell University, Ithaca, NY 14853, USA; 3 R. F. Smith School of Chemical and Biomolecular Engineering, Cornell University, Ithaca, NY 14853, USA

**Keywords:** nanocrystals, self-assembly, monolayers, Langmuir–Blodgett transfer, GISAXS

## Abstract

Grazing incidence small angle X-ray scattering (GISAXS) was used to study the structure and interparticle spacing of monolayers of organic ligand-stabilized iron oxide nanocrystals floating at the air–water interface on a Langmuir trough, and after transfer to a solid support via the Langmuir–Blodgett technique. GISAXS measurements of the nanocrystal arrangement at the air–water interface showed that lateral compression decreased the interparticle spacing of continuous films. GISAXS also revealed that Langmuir–Blodgett transfer of the nanocrystal layers to a silicon substrate led to a stretching of the film, with a significant increase in interparticle spacing.

## 1. Introduction

Langmuir–Blodgett (LB) transfer is the classic method of transferring monolayers at the air–water interface to solid substrates in a layer-by-layer fashion [1,2,3,4]. Essentially, all materials that can be suspended at the air–water interface can be deposited this way [4,5,6]. In fact, a large variety of nanomaterials, including carbon nanotubes, nanocellulose, proteins, and viruses have been recently prepared and transferred via the LB technique [7,8,9,10,11,12,13,14,15]. Here we will focus specifically on ligand-stabilized nanoparticles that can be suspended at the air–water interface [16,17,18,19,20,21,22,23]. Heath and collaborators found that under compression, gold and silver nanocrystal layers can undergo a metal-to-insulator transition in their optical properties [24,25]. More recently, plasmonic effects causing a color change in the compressed gold nanocrystal monolayer were reported [26]. The first systematic in situ structural studies of nanoparticle Langmuir layers with X-ray reflectivity and grazing incidence small-angle X-ray scattering (GISAXS) were reported by Pershan and coworkers [27] who found an irreversible double-layer formation when the films were compressed above a critical limit. These results were corroborated in a recent study by Vegso et al. [28]. In contrast, Viccaro and coworkers [29] as well as Sanyal and coworkers [30] found a reversible wrinkling or buckling of the nanocrystal monolayer. These different findings can be reconciled, though, as long as nanoparticle layers are not compressed to the point of multilayer formation, the compression of the layer remains reversible.

In our previous work we were concerned with preparing monolayers of magnetic nanoparticles via LB transfer or, in a variation of the Langmuir–Schaefer method, to transfer the layer to a PDMS stamp, and successive microcontact printing [31]. In our follow-up work we quantitatively analyzed monolayers [32] and multilayers [33] produced via LB transfer with GISAXS within the quasi-kinematic scattering approximation. In the present work we investigated the optimum conditions for LB transfer of nanocrystals as well as comparing the compressed Langmuir layer with the transferred nanocrystal monolayer using real-time in situ X-ray scattering.

## 2. Materials and Methods

### 2.1. Materials and Supplies

All chemicals were used as received. Iron pentacarbonyl (Fe(CO)_5_, 99.999%) and oleic acid (99%) were purchased from Sigma-Aldrich (St. Louis, MO, USA). Ethanol (ACS grade), 2-propanol (ACS grade), acetone (ACS grade), and chloroform (ACS grade) were purchased from Fisher Scientific (Pittsburgh, PA, USA). Dioctylether (>97%) was purchased from Fluka Chemicals (Morris Plains, NJ, USA). Deionized water (DI-H_2_O) was used in all aqueous preparations.

### 2.2. Iron Oxide Nanocrystal Synthesis

Monodisperse oleic acid-stabilized Fe_2_O_3_ nanocrystals of 7 nm diameter were prepared on a Schlenk line, as previously described [34]. In a 25 mL three-neck flask, 10 mL of dioctyl ether and 1.44 μL (4.56 mmol) of oleic acid were heated to 100 °C under N_2_ flow at atmospheric pressure. Next, 0.2 mL (1.52 mmol) of Fe(CO)_5_ was injected into this solution and the temperature was raised to 300 °C. The solution was refluxed for 1 h before removing the heating mantle and allowing the reaction flask to cool to room temperature. The flask was then opened to air for 30 min to oxidize the as-made Fe nanocrystals. The reaction solution was cleaned up by first centrifuging for 5 min at 8000 rpm (8228 g). The precipitate, consisting of poorly functionalized nanocrystals, was discarded. A total of 5 mL of ethanol was then added to the supernatant and this mixture was centrifuged for 10 min at 8000 rpm (8228 g) to precipitate the nanocrystals. The clear and colorless supernatant was discarded. The nanocrystals were further purified by redispersing in 1 mL of hexane followed by the addition of 2 mL of ethanol as an antisolvent and then centrifuged at 8000 rpm (8228 g) for 5 min. The precipitate was collected and the purification procedure was completed two more times before dispersing the nanocrystals in chloroform.

### 2.3. Nanocrystal Monolayer Formation

Langmuir films of Fe_2_O_3_ nanocrystal monolayers for GISAXS measurements were prepared using a KSV mini trough System 2 enclosed in a plexiglass cabinet. Pure DI-H_2_O was used as the subphase. The trough and barriers were thoroughly cleaned with ethanol and rinsed with DI-H_2_O before the formation of each monolayer. Nanocrystals were spread onto the water surface by depositing small droplets of Fe_2_O_3_ nanocrystals dispersed in chloroform (0.5 mg/mL) via a 100 µL microsyringe (Hamilton Company, Reno, NV, USA). Small droplets were formed at the tip of the syringe and carefully brought into contact with the water surface to ensure that the droplet did not penetrate the water surface and sink to the bottom of the trough. A total of 300 µL of the nanocrystal dispersion was deposited onto the water surface. After allowing 10 min for complete evaporation of the solvent, the LB trough was compressed at 10 mm/min to the desired surface pressure. Surface pressure-area isotherms were recorded using a platinum Wilhelmy plate (KSV Instruments, Helsinki, Finland, product no. 51066) connected to a KSV film balance.

LB films of Fe_2_O_3_ nanocrystal monolayers were transferred from the air–water interface to 15 mm × 15 mm silicon substrates (p-type silicon test wafers with native oxide by NOVA Electronic Materials, Flower Mound, TX, USA) via a vertical lift-off procedure. The silicon substrate was placed vertically below the water surface prior to deposition of the nanocrystals and compression of the film. After depositing the nanocrystals and compressing the film to the desired surface pressure, the substrate was retrieved at a rate of 1 mm/min. The desired surface pressure was maintained during film transfer by allowing the KSV trough software to move the barriers as needed via feedback from the Wilhelmy balance. After each monolayer was transferred to a silicon substrate, the LB trough was thoroughly cleaned with ethanol and DI-H_2_O before the procedure was repeated to create a new monolayer. All silicon substrates were cleaned by rinsing with DI-H_2_O, acetone, and 2-propanol followed by 10 min UV-ozone cleaning (Jelight Company, Irvine, CA, USA, model 42) prior to use.

For in situ grazing-incidence small angle X-ray scattering (GISAXS) measurements, a smaller Langmuir trough was designed to fit within the dimensions of the GISAXS stage on the D1 beamline at the Cornell High Energy Synchrotron Source (CHESS). The Teflon trough was machined from a single block of Teflon having the dimensions of 56 mm L, 56 mm W, 40 mm H. A depression measuring 50 mm by 50 mm with a 2 mm depth and 3 mm wide lip around the edge was machined into one face to contain the water. A moveable barrier (10 mm H, 10 mm W, 60 mm L) was machined from a separate piece of Teflon. In situ compression of the film was achieved by using a syringe pump (KD Scientific, Holliston, MA, USA) with a push rod set in the syringe holder to move the barrier at a calibrated speed. The trough was cleaned with ethanol and rinsed with DI-H_2_O before each use. DI-H_2_O was used as the subphase and 40–150 µL of a 0.5–0.25 mg/mL solution of Fe_2_O_3_ nanocrystals dispersed in chloroform was carefully deposited dropwise onto the water surface.

### 2.4. Materials Characterization

Transmission electron microscopy (TEM) was performed using a FEI Tecnai G2 Spirit BioTWIN (FEI Company, Hillsboro, OR, USA). TEM samples were prepared via drop casting dilute dispersions of nanoparticles in chloroform onto 200-mesh copper grids (Electron Microscopy Sciences, Hatfield, PA, USA). Scanning electron microscopy (SEM) imaging was performed on nanocrystals deposited on silicon substrates using a Zeiss Supra 40 VP field-emission SEM (Carl Zeiss USA, San Diego, CA, USA) at a working voltage of 10 to 15 kV and a working distance of 3 to 6 mm. All TEM and SEM images were acquired digitally.

Grazing incidence small angle X-ray scattering (GISAXS) was performed at the CHESS D1 beamline. Radiation of wavelength λ = 1.252 Å with a bandwidth Δλ/λ of 1.5% was used. The X-ray beam size was 0.5 mm horizontally and 0.2 mm vertically. Scattering patterns were collected using a fiber-coupled CCD camera with 14-bit dynamical range per pixel. All sample to detector distances were calibrated using a silver behenate standard. The sample to detector distance was 957 mm, when scattering images were taken on the nanocrystal films deposited onto silicon substrates. The sample to detector distance was 903 mm, when scattering images were taken at the air–water interface. The incident angle of the X-ray beam was 0.2° for LB films of Fe_2_O_3_ nanocrystals transferred onto silicon substrates. Images were taken with exposure times ranging from 0.1 s to 10 s and processed using FIT2D [35]. For time series during monolayer compression typically 1 s exposures were used to limit radiation damage. Dilute dispersions of Fe_2_O_3_ nanocrystals suspended in hexane were placed in capillary tubes to obtain size distributions of the nanocrystals cores via small-angle X-ray scattering (SAXS). The sample to detector distance for the SAXS setup was 890 mm.

**Figure 1:**
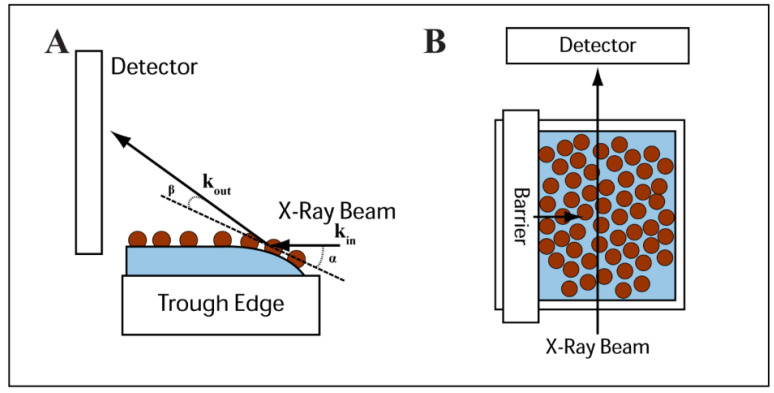
Side view (**A**) and top view (**B**) illustrations of the experimental configuration used for the GISAXS measurements of nanocrystal monolayers floating at the air–water interface of a Langmuir trough. Please note that the effective incident angle of the X-ray beam is exaggerated in panel (**A**).

GISAXS measurements of nanocrystal films transferred to silicon substrates were performed using the standard reflectivity stage. Alignment of the Langmuir trough for in- situ GISAXS measurement of nanocrystals at the air–water interface required a different alignment protocol. First, the standard reflectivity sample holder was replaced with the machined Teflon trough. The center of the small LB trough was machined so that its center corresponded with the center of the reflectivity stage, which provided the same sample to detector distance as measured via the silver behenate standard. Once securely attached to the GISAXS platform, the top surface of the Teflon trough was aligned parallel with the X-ray beam. Pure DI-H_2_O was then added to the trough so that the water height was ~2 mm above the Teflon edge which provided a flat surface to scatter off. A small droplet of nanocrystals dispersed in chloroform was then deposited on the water surface. Scans were taken incrementally in the vertical z-direction, until strong scattering from the nanocrystals surface was observed. Figure 1 shows the X-ray scattering geometry used for the in situ GISAXS measurements of Fe_2_O_3_ nanocrystals at the air–water interface.

## 3. Results and Discussion

### 3.1. Langmuir–Blodgett Film Formation

**Figure 2:**
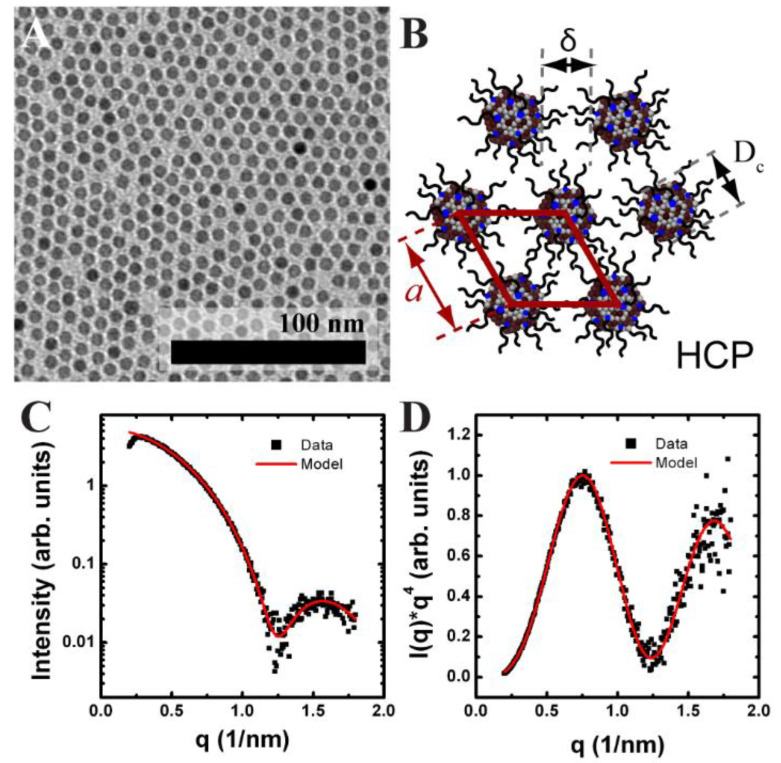
Nanocrystal monolayer characterization. (**A**) TEM of a Fe_2_O_3_ nanocrystal monolayer. (**B**) Schematic of a hexagonal lattice of oleic acid coated nanocrystals with edge-to-edge spacing (*δ*), center-to-center spacing (*a*), and crystalline core (*D_c_*) defined. (**C**,**D**) Solution phase SAXS of Fe_2_O_3_ nanocrystals in hexane. (**C**) Scattering intensity vs. *q* and (**D**) Associated Porod plot of for Fe_2_O_3_ nanocrystals. The experimental data (■) and best fit (solid line) are shown. From the best fit, the core diameter and standard deviation was determined to be 7.14 ± 0.54 nm.

**Figure 3:**
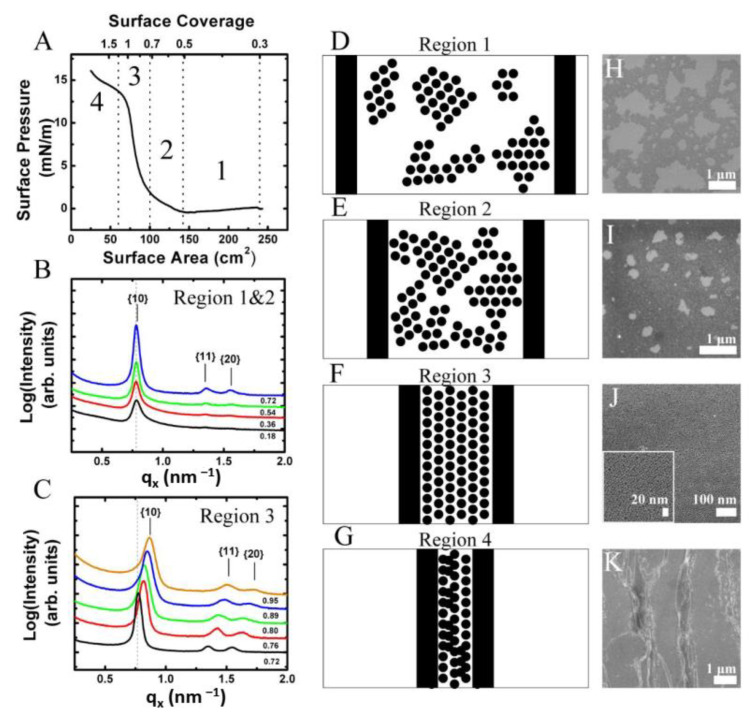
Nanocrystal LB films deposited at different surface pressures. (**A**) Surface pressure–area isotherm of 7.14 ± 0.54 nm oleic acid stabilized Fe_2_O_3_ nanocrystals on a Langmuir–Blodgett trough. The film was compressed at 10 mm/min. A total of 300 µL of a 0.5 mg/mL dispersion of Fe_2_O_3_ nanocrystals dispersed in chloroform was deposited onto an area of 250 cm^2^. Vertical dashed lines mark the regions of the isotherm corresponding to the gas (1), liquid (2), solid (3), and collapse (4) phases. The upper *x*-axis denotes the surface coverage of Fe_2_O_3_ nanocrystals. (**B**) GISAXS performed on Fe_2_O_3_ nanocrystals films at the air–water interface over a surface coverage range of 0.18–0.72 which correspond to gas and liquid phases (regions 1 and 2) of the isotherm. (**C**) GISAXS scattering performed on Fe_2_O_3_ nanocrystals films at the air–water interface during compression over a surface coverage range of 0.72 to 0.95 which correspond to solid phase (region 3) of the isotherm. The graphs are projection integrations of GISAXS scattering images taken at various surface coverages on the air–water interface. The edge-to-edge separation between nanocrystals remains at 2.1 nm as the surface coverage increases from 0.18 to 0.72 during nanocrystal deposition and decreases from 2.1 nm to 1.3 nm over a surface coverage of 0.72 to 0.95. (**D**–**G**) are schematic illustrations of nanocrystal assembly at the air–water interface within the regions denoted in panel (**A**). A top-down view of the LB trough is depicted. Black circles correspond to Fe_2_O_3_ nanocrystals and vertical black bars correspond to the trough barriers. For simplicity, the ligands on the surface of the nanocrystal were not drawn. (**H**–**K**) SEM images of vertically transferred films at surface pressures of (**H**) 0 mN/m, (**I**) 1 mN/m, (**J**) 12 mN/m, and (**K**) 16 mN/m. SEM images are placed adjacent to corresponding film morphologies depicted in images (**D**–**G**).

Hexagonally ordered monolayers of oleic acid-stabilized Fe_2_O_3_ nanocrystals were assembled using an LB trough. Figure 2A shows TEM images of the 7.14 ± 0.54 nm diameter Fe_2_O_3_ nanocrystals used. The diameter and diameter distribution of the Fe_2_O_3_ nanocrystals was determined from solution SAXS (Figure 2C,D). Figure 3A shows typical surface pressure-area isotherms of the Fe_2_O_3_ nanocrystals. The isotherms showed two phase transitions before the film buckled, causing the formation of multilayer stripes. The Fe_2_O_3_ nanocrystals started in the gas phase, which comprised nanocrystal islands each having hexagonal order (Figure 3A, region 1). Further compression of the film led to the first phase transition from the gas to liquid state, which can be seen on the isotherm at a surface area of 144 cm^2^ (surface coverage of 0.5) (Figure 3A, region 2). SEM of the LB film transferred to a silicon substrate in this compression region showed the gathering of hexagonally ordered nanocrystal islands and a reduction in the large void space between them.

Further compression of the film resulted in a rapid increase in surface pressure and transition from the liquid state to the solid phase which can be seen at a surface area of 87 cm^2^ (surface coverage of 0.82) (Figure 3A, region 3). Further compression of the LB film resulted in buckling of the film causing the formation of multilayer wrinkles, which were observed via SEM inspection of the vertically transferred nanocrystal films. The buckling transition was also observed by a shoulder in the isotherm at a surface area of 72 cm^2^ (Figure 3A, region 3). The point in the surface pressure-area curve with the maximum slope was defined to correspond to a surface coverage of 1. Then the surface coverage is given as *A_min_*/*A* where *A_min_* is the smallest surface and *A* is the current surface area as given by the position of the barriers.

A continuous monolayer was transferred to a silicon substrate within the solid phase region at a surface pressure of 12 mN/m corresponding to a surface coverage of 0.98, i.e., just below the onset of the wrinkling transition. The continuous nanocrystal monolayer comprised multiple hexagonally ordered nanocrystal grains. The monolayers were free of large voids and contained only small holes on the order of 20 to 50 nm and defects such as particle vacancies resided predominantly at grain boundaries (Figure 3J). The LB film evolution and shape of the isotherms were consistent with the compression of hexagonally ordered nanocrystal domains observed for both the LB films of bare oleic acid/oleylamine-capped FePt nanocrystals and octadecyltrimethoxy silane-capped FePt@SiO_2_ core-shell nanoparticles as we previously reported [31].

### 3.2. In Situ GISAXS of Fe_2_O_3_ Nanocrystals at the Air–Water Interface during Nanocrystal Deposition

In situ GISAXS experiments were performed at CHESS D1 beamline using a smaller home-built LB trough, in order to study the edge-to-edge separation of the 7 nm diameter Fe_2_O_3_ nanocrystals during compression. Figure 1 depicts the experimental setup showing the side and top view of the trough with respect to the X-ray beam.

**Figure 4:**
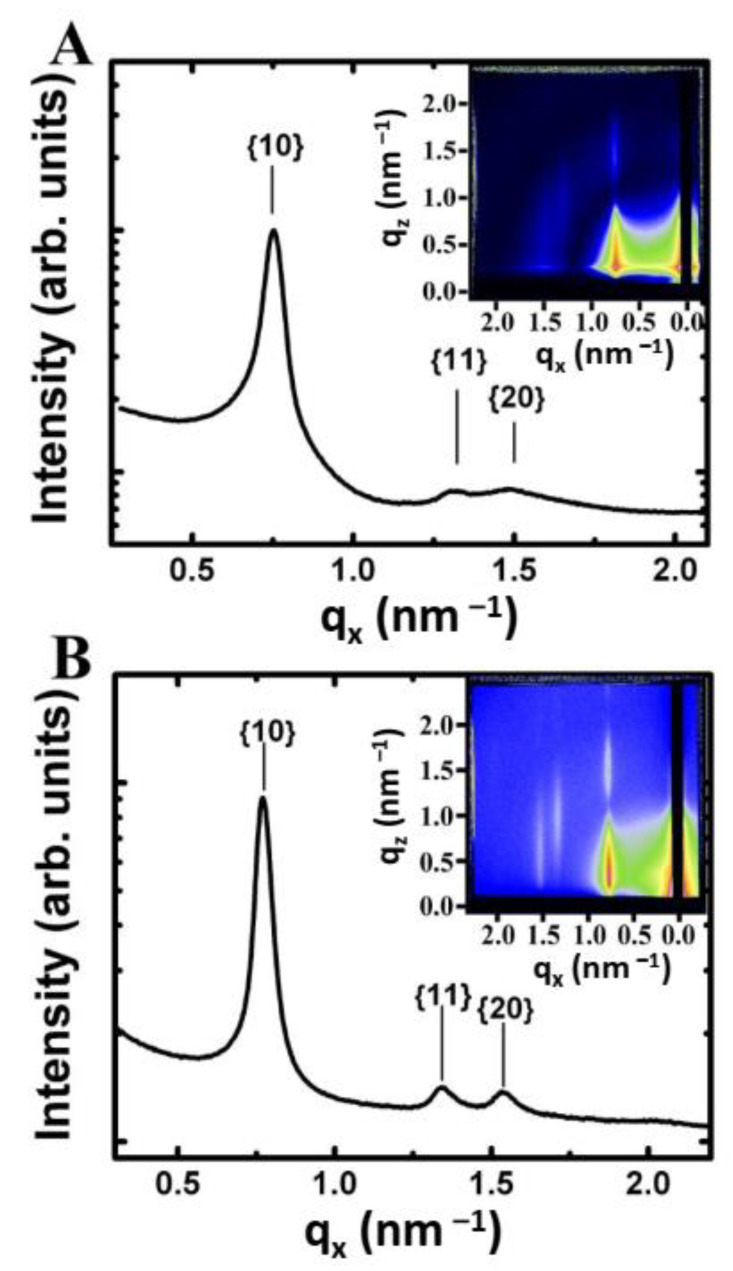
Comparison of GISAXS scattering images for Fe_2_O_3_ nanocrystals on a silicon substrate vs. at the air–water interface. (**A**) GISAXS scattering pattern (inset) for a monolayer of 7.14 nm Fe_2_O_3_ nanocrystals on a silicon substrate transferred at a surface coverage of 0.89. (**B**) GISAXS scattering pattern (inset) for a monolayer of 7.14 nm Fe_2_O_3_ nanocrystals at the air–water interface taken at a surface coverage of 0.72. Both images are plotted on a logarithmic false-color scale where blue corresponds to an intensity of 10 and red to 10,000 in arbitrary units. The associated plots are projection integrations onto the *q_x_*-axis of the scattering image. The lattice row indices of a hexagonal monolayer are indicated on the projection integration.

Fe_2_O_3_ nanocrystals were added drop-wise to the air–water interface of the in situ LB trough in incremental aliquots of 10 µL for the 7 nm diameter Fe_2_O_3_ nanocrystals. Figure 3 shows the GISAXS scattering projection integrations of nanocrystals deposited on the air–water interface after each incremental deposition. GISAXS scattering images of the film, even at low nanocrystal surface coverage, revealed the presence of scattering peaks indicating the formation of nanocrystal islands with hexagonal order at the air–water interface [32]. The scattering was weak and the higher order {11} and {20} Bragg rods were barely visible. With increasing surface coverage (i.e., number of scatters), the 7 nm diameter Fe_2_O_3_ nanocrystals showed an increase in scattering intensity and the observance of the higher order Bragg rods indicating long range order (Figure 4). The ratio of the Bragg rod peak positions versus the primary peak for the Fe_2_O_3_ nanocrystals at the air–water interface followed a sequence of 1, $\sqrt{3}$, $\sqrt{4}$ confirming the formation of hexagonally ordered domains as previously observed via SEM in the corresponding LB films.

The peak position of the first-order {10} Bragg rod for the Fe_2_O_3_ nanocrystal LB film in Figure 3 corresponds to *q_x_* = 0.78 nm^–1^ and yields a *d*-spacing of 8.0 nm. Given that the monolayer forms a hexagonal lattice, the center-to-center spacing (*a*) between two nanocrystals can be determined from simple geometrical arguments (*a* = 2/$\sqrt{3}$ *d*) (Figure 2B). The edge-to-edge spacing (*δ*) between nanocrystals is calculated by subtracting the nanocrystal core diameter (*D_c_*) from the center-to-center spacing (*δ* = *a*–*D_c_*) (see Figure 2B). For the scattering image shown in Figure 4B for 7 nm diameter hexagonally ordered Fe_2_O_3_ nanocrystals, the center-to-center spacing is 9.24 nm and edge-to-edge spacing is 2.1 nm. A similar analysis was performed on all of the GISAXS scattering images as the nanocrystal surface coverage increased from 0.18 to 0.72. The edge-to-edge separation remained at 2.10 nm. In this regime, no change in the edge-to-edge separation was expected as the water surface contained nanocrystal islands separated by large amounts of void space.

### 3.3. In Situ GISAXS of Fe_2_O_3_ Nanocrystals at the Air–Water Interface during Film Compression

The film of nanocrystals at the air–water interface was compressed to a higher surface coverage to determine changes in the edge-to-edge separation between nanocrystals. Starting at a surface coverage of 0.72, the nanocrystal film was compressed at a controlled rate using a Teflon barrier. The compression region is denoted as region 3 on the isotherm in Figure 3A. The film was compressed at a rate of 0.5 mm/min and GISAXS scattering images were taken approximately every 10 s to monitor the compression of the film. The film was compressed to a final surface coverage of 1.2. However, only scattering images taken between a surface coverage of 0.72 and 0.95 were available. This smaller surface coverage window was due to the interaction of the X-ray beam with water meniscus at the edge of the barrier which significantly tilted the Bragg rods and eventually blocked the beam as the barrier moved across the center of the in situ LB trough.

**Figure 5:**
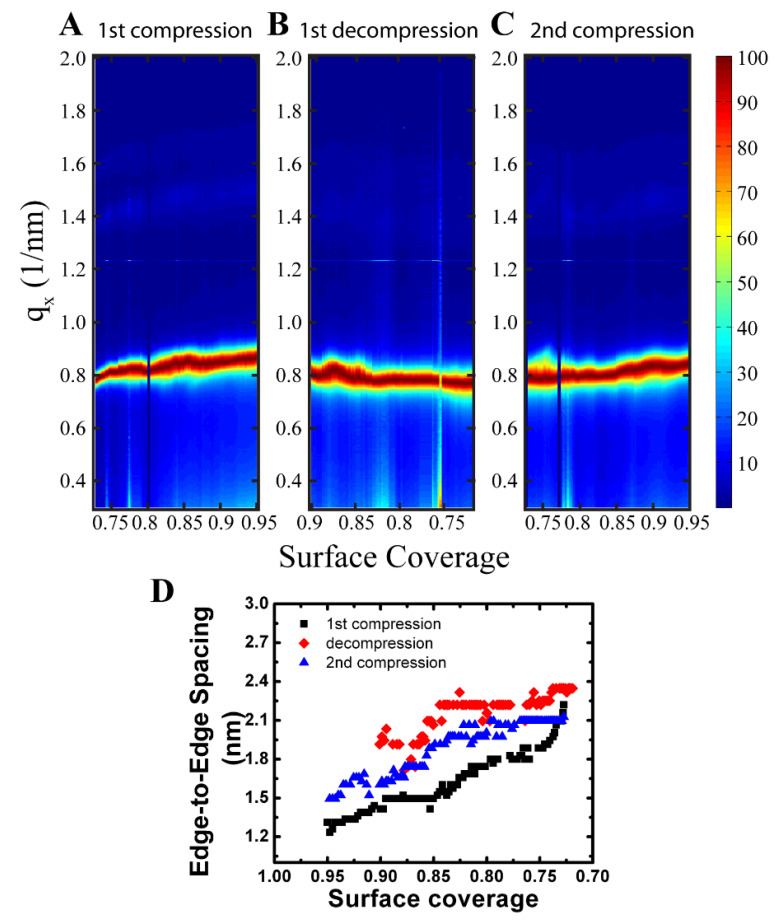
Contour plots of GISAXS scattering projection integrations taken during first compression (**A**), first decompression (**B**), and second compression (**C**) cycles of 7.14 nm diameter Fe_2_O_3_ nanocrystals at the air–water interface. The film was compressed and then decompressed at 0.5 mm/min with scattering images taken approximately every 10 sec. The film was compressed from a surface coverage of 0.72 to 1.2, however scattering beyond a surface coverage of 0.95 (not shown) resulted in blocking of the X-ray beam by the moving barrier. (**D**) Plot of edge-to-edge separation vs. surface coverage during the first compression (■), first decompression (♦), and second compression (▲) cycles. Calculation of the edge-to-edge separation was determined from the peak position of the {10} Bragg rod.

During the compression, a smooth shift in the *q_x_* position to higher *q*-values, and hence lower *d*-spacings, was observed for the Bragg rods in the GISAXS scattering images (Figure 3C). The relative peak positions of the {10}, {11}, and {20} rods also shifted equally maintaining the hexagonally close-packed 1, $\sqrt{3}$, $\sqrt{4}$ peak position sequence (Figure 3C). Extraction of the edge-to-edge spacing from the *q_x_* peak position of the {10} Bragg rod as a function of surface coverage during the first compression is shown as the black squares in Figure 5D. Compression of the film from a surface coverage of 0.72 to 0.95 resulted in a steady decrease in the edge-to-edge spacing from the starting equilibrium value of 2.1 nm to 1.3 nm (~0.8 nm) indicating compression of the ligand layer between the nanocrystals. An increase in scattering intensity near the beam stop was also observed which was attributed to forming a more continuous scattering layer as the nanocrystals moved closer together.

At equilibrium, the edge-to-edge separation between nanocrystals has been observed in many systems to be on the order of one ligand length instead of two, if the ligands were fully extended [27,36,37]. For the system presented here, the edge-to-edge spacing was 2.1 nm at the start of compression which is approximately the length (*l*) of an oleic acid molecule (2.2 nm). The small spacing between particles suggests displacement of the ligands occurred upon contact of the nanocrystals. The ability to compress the ligand layer was derived from the packing density of ligands on the surface of the nanocrystal. Small diameter nanocrystals have a much higher surface curvature resulting in a higher free volume available to the ligand allowing it to deform as it interacts with other nanocrystals in the film. Given the footprint (*f*) of oleic acid is 0.41 nm^2^ and the diameter of the nanocrystal is 7.14 nm, the number of ligands on the surface of each nanocrystal can be estimated as about 390. The volume available to each ligand, assuming no interaction between nanocrystals, was 1.6 nm^3^/oleic acid. The volume per ligand was estimated by dividing the volume of a ligand layer, 2.2 nm thick on the surface of a nanocrystal, by the number of ligands per nanocrystal. 

When the nanocrystals were deposited from the solution onto the air–water interface, they assembled into a hexagonal lattice with edge-to-edge spacing of 2.1 nm. Based on this configuration, the volume available to each ligand in the interstitial space was 0.94 nm^3^/oleic acid which agreed well with the volume of an oleic acid molecule modeled as a cylinder, *f·l* = 0.90 nm^3^/oleic acid, indicating ligand flexibly allows for complete space filling of the void space in the lattice. Compression of the film to an edge-to-edge spacing of 1.3 nm resulted in a decrease in volume per ligand to 0.60 nm^3^/oleic acid.

This result seems counterintuitive given the effective volume of an oleic acid molecule is 0.9 nm^3^/oleic acid, however, it can be explained by examining the ligand structure. Oleic acid is a C18 carbon chain molecule with a double bond between carbon atoms 9 and 10 resulting in a cis configuration (i.e., banana shaped) which reduces the linearity of the molecule and interferes with the close-packing between hydrocarbon chains. Oleic acid’s linear analog is stearic acid which has a footprint of 0.20 nm^2^, length of 2.5 nm, and volume of 0.5 nm^3^/stearic acid, which is more representative of the physical volume of an oleic acid molecule [38,39]. Given this value, the observed decrease in the interparticle spacing during film compression was likely caused by an increase in packing efficiency of the oleic acid molecules on the surface of the nanocrystal caused by compression of the film. For comparison, the volume per oleic acid molecule in a ß_1_ phase crystal is 0.45 nm^3^/oleic acid corroborating the increase in packing efficiency during compression [40].

Ligand compression on the nanocrystal surface displayed interesting similarities to the compression of a surfactant monolayer of pure oleic acid. Unlike a functionalized nanocrystal, which sits at the air–water interface with the hydrophobic ligand tails interacting with the water phase, surfactant monolayers sit on the air–water interface with their polar head groups in contact with the water and hydrophobic tail extending away from the water surface. In both cases, the hydrophobic ligand tails are free to interact with their neighbors which gives rise to the changes observed in the surface pressure-area isotherms. When deposited onto a water surface, oleic acid molecules are known to form an expanded monolayer due to the reduction in cohesion between ligand chains resulting from the cis configuration of the hydrophobic tail [41].

This is analogous to the ligand state on the surface of the nanocrystal at the start of compression. Compression of the film by the barrier forces the ligand tails to interact and decreases the volume available which is analogous to the reduction in ligand volume observed on the surface of the nanocrystal during compression. This interaction can be characterized by determining the compressibility of both films. Surprisingly, both the oleic acid-capped nanocrystals and the pure oleic acid monolayer had similar compressibilities, 0.02 m/mN (nanocrystal) vs. 0.022–0.0175 m/mN (surfactant film) [17,42]. This result indicates that ligand tail interactions and ligand morphology play an important role in the compression of nanocrystal films. It is expected for linear, saturated ligands, such as stearic acid, which are known to form condensed monolayers at low surface pressures, to not display such high ligand compression because the initial packing of the nanocrystals will result in a free ligand volume on the order of the capping ligand volume [42].

In situ GISAXS compression experiments performed on nanocrystals with linear ligands such as 6 nm dodecanethiol capped gold, 2 nm dodecanethiol capped gold, and 2.2 nm mercaptohexadecanoic acid capped gold on a LB trough confirmed this statement as no compression of the ligand shell was observed [27,29,30]. The previously mentioned GISAXS results conflicted with the results of Collier et al. who attributed a metal-to-insulator transition with a decrease in edge-to-edge spacing of 2.7 nm hexanethiol capped silver nanocrystals compressed on an LB trough [24]. Their results may be explained by the reduced length of the hexanethiol capping ligand used (C6 vs. C12). Shorter ligands are known to have weaker chain–chain interactions resulting in expanded films on the water surface that need higher surface pressures to transition to a condensed monolayer [24]. It is believed that this transition is what causes the decrease in interparticle spacing and metal-to-insulator transition. In the same study the ligand length was increased to a C10 chain (decanethiol) and no metal-to-insulator transition was observed due to the increased chain–chain interactions which results in a more condensed ligand layer on the surface of the nanocrystal leaving little room for compression and thus not reaching the critical density for the metal-to-insulator transition.

### 3.4. In Situ GISAXS Characterization of Fe_2_O^3^ Nanocrystals at the Air–Water Interface during Film Expansion

GISAXS images were also taken at the same film as it decompressed from a surface coverage 1.2 to 0.72 at 0.5 mm/min (Figure 5B). The edge-to-edge separation with decompression is shown in Figure 5D as the red diamonds. The edge-to-edge separation relaxes to the starting edge-to-edge separation of 2.2 nm at a much higher surface coverage of 0.84 causing hysteresis in the measurement. The observed hysteresis may be caused by buckling and wrinkling of the nanocrystal film upon compression as seen by the surface pressure-area isotherm (Figure 3A). The maximum surface coverage reaches a value of 1.2 for the in situ GISAXS experiments, which is beyond the buckling point of the film (Figure 3A). Wrinkling of the nanocrystal film causes the area taken up by the nanocrystals on the air–water interface to decrease due to the formation of multilayers, thus reducing the surface coverage of nanocrystals on the air–water interface. As the film is decompressed, the change in surface coverage occurs quickly resulting in a faster return to the equilibrium edge-to-edge separation of 2.2 nm. Since the film reached a surface coverage of 1.2, approximately 16% of the film was converted to a multilayer. Using this value, it is expected for the nanocrystal spacing to relax to a value of 2.1 nm at a surface coverage of 0.86 which agreed favorably with the experimental result of 0.84 obtained. If the film is compressed again, it is expected for the edge-to-edge spacing to reach a value of ~1.6 nm at a surface coverage of 0.95.

To confirm buckling of the film, a second compression of the nanocrystal film was performed at a barrier speed of 0.5 mm/min with GISAXS images taken approximately every 10 s (Figure 5C). The change in edge-to-edge separation of the second compression is shown in Figure 5D as the blue triangles and follows very closely to the trend in the decompression cycle. The starting edge-to-edge separation is 2.1 nm at a surface coverage of 0.72 and remained unchanged until a surface coverage of 0.82 where further compression results in a continuous decrease in the edge-to-edge separation. The transition agreed favorably with the transition of the decompression cycle which occurred at a surface coverage of 0.84. At a surface coverage of 0.95, the edge-to-edge spacing was 1.5 nm which agreed well with predicted value of 1.6 nm, thus the shift in the onset of ligand compression was due to buckling of the film and the formation of multilayers. SEM of the nanocrystal film transferred to a silicon substrate also confirmed the formation of multilayers (Figure 3K) which was consistent with film buckling. The results indicated that the compression of the ligand layer on the surface of the nanocrystal was reversible and stemmed from the cis configuration of the oleic acid molecule which preferred to be in an expanded rather that closed packed state.

### 3.5. GISAXS Characterization of Fe_2_O_3_ Nanocrystal Monolayers Transferred onto Silicon Substrates Using a Langmuir–Blodgett Trough

GISAXS scattering images of 7 nm Fe_2_O_3_ nanocrystal LB films vertically transferred to silicon substrates at different nanocrystal surface coverages were taken at CHESS using an incident angle of 0.2°. The same 7 nm diameter Fe_2_O_3_ nanocrystals used for the in situ GISAXS measurements of Fe_2_O_3_ nanocrystals at the air–water interface were also used to prepare nanocrystal films on silicon substrates. Additionally, both the preparation of LB films on silicon substrates as well as the in situ GISAXS measurements of the Fe_2_O_3_ nanocrystals at the air–water interface were obtained within a week of nanocrystal synthesis and purification in order to minimize any changes in nanocrystal behavior due to ligand desorption from the Fe_2_O_3_ nanocrystal surface over time [31].

The inset of Figure 4A shows a representative GISAXS detector image from a monolayer of Fe_2_O_3_ nanocrystals transferred to a silicon substrate at a surface coverage of 0.89. The orientation of the substrate retrieval direction was perpendicular to the direction of the X-ray beam. The graph shows an *q_x_*-projection integration of the detector image and reveals three distinct peaks with a ratio of their *q_x_* peak positions to the primary peak following a sequence of 1, $\sqrt{3}$, $\sqrt{4}$ indicating hexagonal order which was consistent with in situ GISAXS measurements (Figure 3). Subtle differences in the GISAXS scattering pattern of the transferred LB films on silicon and the in situ prepared monolayers at the air–water exist (Figure 4B). The scattering peaks were sharper in the in situ measurement as seen by comparison of the *q_x_*-projection integrations shown in Figure 4A (ex situ) and Figure 4B (in situ). The higher order peaks corresponding to the {11} and {20} rows were also more intense and clearly separated in comparison to the transferred films on silicon, where the two weak peaks appear to overlap (Figure 5A vs. Figure 5B).

**Figure 6:**
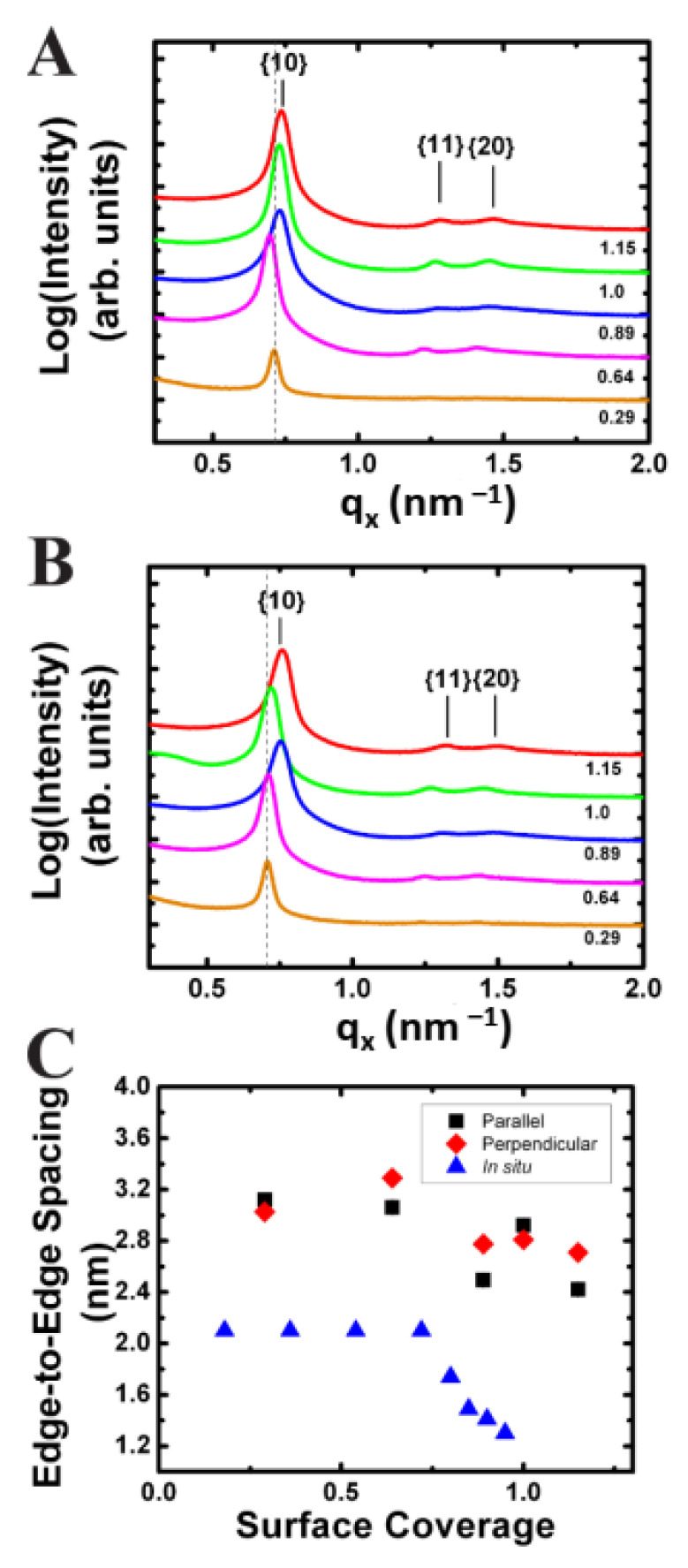
Projection integrations of GISAXS scattering images for 7.14 nm Fe_2_O_3_ nanocrystal monolayer films deposited onto silicon substrates at various nanocrystal surface coverage with the X-ray beam oriented both perpendicular (**A**) and parallel (**B**) to the substrate retrieval direction. (**C**) Plot of the edge-to-edge spacing vs. surface coverage for nanocrystal films transferred to silicon substrates with the X-ray beam (■) perpendicular and (♦) parallel to the dipping direction. For comparison, the edge-to-edge spacing for nanocrystals compressed at the (▲) air–water interface is also plotted.

In addition, GISAXS scattering images were taken of Fe_2_O_3_ nanocrystals transferred to silicon substrates at various stages of compression with the X-ray beam both parallel and perpendicular to the dipping direction (Figure 6A,B). The edge-to-edge spacing versus surface coverage is plotted in Figure 6C for both orientations of the X-ray beam with respect to the substrate retrieval direction. An overall decrease in edge-to-edge spacing of 0.7 nm, from 3.1 nm to 2.4 nm, was observed with increasing surface pressure.

**Figure 7:**
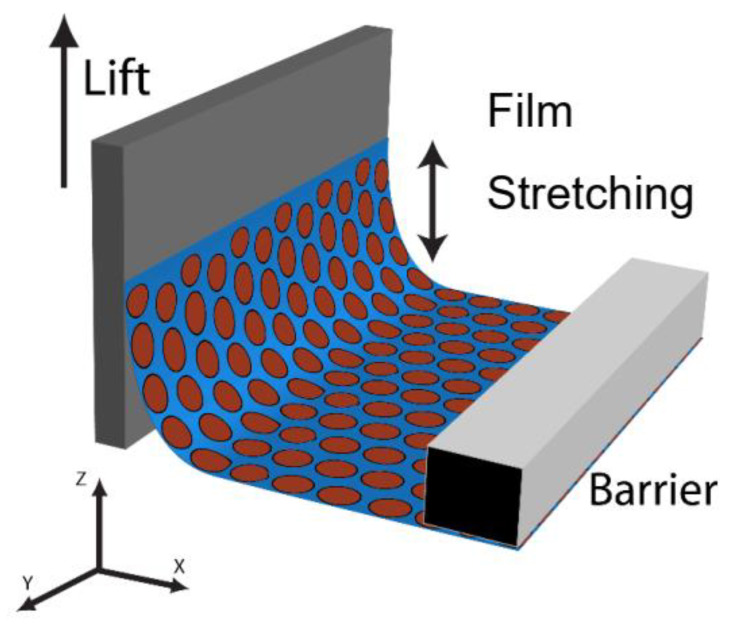
Langmuir–Blodgett vertical transfer of a nanocrystal monolayer from the air–water interface onto a solid substrate.

Interesting to note are the small differences in the edge-to-edge spacing with respect to the orientation of the X-ray beam and substrate retrieval direction (Figure 6A,B). Nanocrystal monolayers consist of multiple hexagonally ordered grains having random orientations with respect to the X-ray beam, therefore these powder films are not expected to have any rotational dependence on their scattering peak positions. Analysis of the films with the dipping direction both perpendicular and parallel to the X-ray beam indicated that on average the parallel orientation had a smaller edge-to-edge separation in comparison to the perpendicular orientation. For example, at a surface pressure of 7 mN/m, corresponding to a surface coverage of 0.9, the perpendicular orientation had an edge-to-edge separation of 2.76 nm compared to 2.49 nm observed in the parallel orientation (Figure 6C). The difference is significant given the resolution of the detector in this region was 0.03 nm. The result indicated expansion of the hexagonal lattice in the *z*-direction caused by slipping of the nanocrystals rows as the film was transferred onto the substrate (Figure 6C). The nanocrystal monolayer was confined to the width of the silicon substrate, leaving little room for the film to expand. In contrast, there was no such constraint in the direction of the LB transfer, and hence the transferred films were slightly expanded. (Figure 7).

Transfer of the monolayer begins at the three-phase contact line between the substrate, water, and air [43,44,45]. A nanocrystal contact line is first pinned at the interface and as the substrate is withdrawn, the continuous deposition of the monolayer is governed by the interaction of the particles with the substrate, interparticle interactions, substrate withdrawal speed, and the interfacial force applied to the substrate [44,45,46,47]. Substrates were withdrawn from the water subphase at a rate of 1 mm/min for the transfer of all LB films to ensure a smooth moving meniscus in order to minimize stick-slip at the three-phase contact line and water entrainment between the nanocrystal monolayer and substrate [44]. Despite these precautions, the small changes observed here indicate stick-slip can occur at length scales on the order of 0.3 nm that were difficult to accurately measure via SEM and TEM measurements. For stick-slip film structures, typically large macroscopic changes in the nanocrystal lattice (~50 µm) are observed such as the periodic line patterns demonstrated by Huang et al. [46].

Comparison of the edge-to-edge separation measurements taken from the LB films on silicon substrates and in situ GISAXS measurements at the air–water interface revealed a large change in the edge-to-edge separation upon transfer of the LB films at low surface coverage. On LB films transferred to silicon substrates in the liquid phase at a surface coverage of 0.64, the value of the edge-to-edge separation was 3.3 nm compared to 2.10 nm measured over the surface coverage range of 0.18–0.72 on the air–water interface. The difference in edge-to-edge separation indicated a slip of 1.2 nm between the {10} rows giving further evidence of nanocrystal slipping under the vertical deposition conditions for films taken at low surface coverage. Comparison of the edge-to-edge separation to the value obtained with the parallel X-ray beam and retrieval orientation for transferred films shows a slightly smaller change in edge-to-edge separation of 0.9 nm (2.1 nm (in situ) vs. 3.0 nm (ex situ)) indicating some expansion in the *y*-direction occurred during transfer. This may be induced by the natural parabolic curvature of the water meniscus when in contact with the substrate causing the monolayer to expand in the *y*-direction [47,48,49,50].

### 3.6. Nanocrystal Transfer at High Surface Pressure

The results of decompression indicate the nanocrystal film is in a metastable state and the stored energy in the ligand layer can be easily released, like a spring, with small perturbations of the liquid surface. During transfer, the three-phase contact line causes the water subphase to bend and locally increases the surface area available to the nanocrystal film allowing the ligands on the nanocrystal surface to relax before transfer. The increase in local surface area is dependent on the contact angle, surface tension, and lift speed. Under static conditions, the relationship between meniscus height (*h*) and contact angle (*θ*) is given by Equation (1), where *γ* is the surface tension, *ρ_w_* is the density of water, and *g* is gravity [44].

(1) $$h=\sqrt{\frac{2\gamma }{\rho_{w}g}(1-sin\theta )}$$

At low contact angles, the meniscus height increases resulting in an increase in local surface area. Furthermore, it is well known for the contact angle to decrease even further as the substrate is lifted from the water subphase [45]. Low contact angles are desired for the continuous transfer of nanocrystal monolayers, but the resulting local expansion of the water subphase causes the compressed ligand layer on the surface of the nanocrystals to relax resulting in an expansion of the hexagonal lattice. This can be seen by comparing the edge-to-edge separation of films on water at surface coverage of 0.95 to films transferred onto silicon substrate at a surface coverage of 1.0. The edge-to-edge separation on the compressed air–water surface was significantly smaller than that measured for a transferred film on a silicon substrate just before buckling, 1.3 nm vs. 2.8 nm, respectively. At high surface coverage, the film was continuous and the compressibility of the film was low allowing the barrier to efficiently move material towards the substrate. This should alleviate slipping of the film in the *z*-direction, however expansion in an edge-to-edge spacing beyond the equilibrium value of 2.1 nm to a value of 2.8 nm indicates some slipping of the film still occurs.

## 4. Conclusions

We have provided a detailed study of the LB transfer of nanoparticle monolayers to solid substrates, starting from the stages of layer compression to the transferred films. We have shown that GISAXS provides a powerful tool for the quantitative analysis of 2D nanocrystal monolayers at both solid and liquid interfaces. We demonstrated using in situ GISAXS that the edge-to-edge separation of oleic acid-capped 7 nm Fe_2_O_3_ nanocrystals can be decreased via lateral compression of a hexagonally packed monolayer at the air–water interface of a Langmuir–Blodgett trough due to an increase in ligand packing efficiency. Transfer of the films to silicon substrates resulted in an expansion of the hexagonal lattice. We propose that lattice expansion is due to two main mechanisms: (1) slipping caused by weak interparticle interactions and (2) relaxation of the compressed ligand shell due to the local increase in surface area near the substrate. These observations have significant implications on the transfer of 2D nanocrystal monolayers via the vertical-transfer Langmuir–Blodgett technique. For one, the properties associated with the films at the air–water interface, either having optical or metallic behaviors based on proximity of nanocrystals cores, may not be as transferable as expected. Additionally, TEM measurements of ligand spacing in nanoparticle films transferred to solid substrates are not a direct measurement of what may be going on at the trough surface. Finally, relaxation of the film during transfer may occur anisotropically, therefore, relaxation/slipping of nanocrystal monolayers upon Langmuir–Blodgett transfer may not be isotropic in the perpendicular and parallel directions with respect to compression of the films. Taking these details and limitations into account we can state that LB-transfer remains a convenient and versatile method for the transfer of compact nanomaterial monolayers onto solid substrates.

## Data Availability

The original contributions presented in the study are included in the article; further inquiries can be directed to the corresponding authors.
